# Taxonomic revision of the Carpathian endemic Pedicia (Crunobia) staryi species–group (Diptera, Pediciidae) based on morphology and molecular data

**DOI:** 10.3897/zookeys.569.7458

**Published:** 2016-02-26

**Authors:** Avar-Lehel Dénes, Levente-Péter Kolcsár, Edina Török, Lujza Keresztes

**Affiliations:** 1 Hungarian Department of Biology and Ecology, Faculty of Biology and Geology, University Babeș–Bolyai, Clinicilor 5–7, Cluj Napoca, Romania; 2 Interdisciplinary Research Institute on Bio–Nano–Sciences of Babeș–Bolyai University, Treboniu Laurian 42, 400271, Cluj-Napoca Romania; 3 Romanian Academy, Institute of Biology, Splaiul Independenței 296, 060031, București, Romania

**Keywords:** Crunobia, cryptic lineage, new species, re-description, endemism, identification key

## Abstract

Three new species of the genus *Pedicia*, subgenus *Crunobia* (Diptera: Pediciidae) belonging to the *staryi* group are described on the basis of a combination of molecular and morphology datasets, and a key to discriminate between species of the subgenus *Crunobia* is added. Geographic projection of the identified taxa suggests insular-like distribution and shows the importance of the Carpathians as a genetic center which is home to an exceptionally high aquatic diversity in Europe.

## Introduction

The Holarctic genus *Pedicia* Latreille, 1809 is a small taxonomic unit with three subgenera, *Amalopis* Haliday, 1856 (with 12 species group taxa, including 2 subspecies of *Pedicia tenuiloba* Alexander, 1957) and *Crunobia* Kolenati, 1859 (with 16 species). The distribution of these two subgenera is limited within the Palaearctic area, while the remaining subgenus *Pedicia* (with 33 species) extends to the whole Holarctic region (Oosterbroek 2015). The taxonomy of all three subgenera is poorly studied, and recent studies reveal incompletely described biodiversity and important cryptic diversity even from regions that have been better-studied, such as the Western Palaearctic or North America (Ujvárosi and Starý 2003, Ujvárosi and Bálint 2012, Paramonov 2009, Petersen 2006). *Crunobia* was established as a separate genus by Kolenati (1859) for the European widespread species *straminea* (Meigen, 1838) [as *Crunobia schienerii* (Kolenati, 1859)] and later as a subgenus of *Pedicia* by Edwards (1938). The adults belonging to *Crunobia* have important features that are distinct from the features of adults of *Amalopis* or *Pedicia*. For instance, *Crunobia* adults have no conspicuous dark triangles or stripes on the wings, proctiger not sclerotized (membranous) and the gonostylus has only one complicated projection with 2–11 stout black thorns on its dorsal surface (Dienske 1987). Species belonging to this subgenus are present mostly in different mountainous ranges of Europe, with only a few species recorded from Turkey and the Northern Caucasus so far. Only a single species was discovered in Eastern Asia (Alexander 1938, Oosterbroek 2015). However, this species (*Pedicia patens* Alexander, 1938) should be excluded from this group, due to the sharp differences of the male genital structures. Savchenko (1986) recognized two morphologically distinct species groups in *Crunobia* based on the number of chitinous thorns of the dorsal apex of the gonostylus. The most species-rich “*littoralis*” group has only two (three in the case of *Pedicia nielseni*) black spines, while the remaining species belonging to the “*staryi*” group have more than two such projections. The systematic position of the Eastern Asian *Pedicia patens* remains uncertain. The European *Pedicia straminea* (Meigen, 1838) is the most widespread member of the *staryi* species group; the remaining species are endemics with limited range in the Carpathians (*Pedicia apusenica* Ujvárosi & Starý, 2003; *Pedicia lobifera* Savchenko, 1986; and *Pedicia staryi* Savchenko, 1978) and the Balkan Range (*Pedicia spinifera* Starý, 1974). *Pedicia spinifera* was first described from the Rila Mountains, and similar specimens were also later discovered in the Rhodope Mountains in Bulgaria (Kolcsár et al. 2012). These species share many common features with *Pedicia straminea*, but differ because they have a spine in the middle of the interbasis. The remaining three species are microendemics limited to the Carpathians and found in only one or a few mountain enclaves. In the case of *Pedicia staryi* the specimens were first described from a small area in the Ukrainian Carpathians, and were also later discovered in the Eastern and Southern Carpathians (Ujvárosi 2005). The case of *Pedicia lobifera* is similar. First described by Savchenko in 1986, it has long been considered a species restricted to the Ukrainian Carpathians, but was later discovered in the Eastern Carpathians, Romania, as well (Kolcsár et al. 2012). The most problematic situation is with the newest member of the group, *Pedicia apusenica* (Ujvárosi and Starý 2003), which has been found in an isolated region of the Carpathians, the Apuseni Mountains. Initially considered a sister species of *Pedicia spinifera* (due to very limited material available at the time), the distinctive characters identified in the original description did not exemplify a clear delineation between *Pedicia staryi* and *Pedicia apusenica*. A revision of the entire species group was suggested on the basis of an intensive sampling in the whole distribution area. Later, an exceptionally high molecular variability was detected within the *Pedicia staryi* group, hence the emerging need for a comprehensive revision (Dénes et al. 2015).

## Material and methods

Adult specimens were collected between 2003 and 2015 (Suppl. material 1) using sweep nets and were stored in 96% ethanol. The morphological characteristics of the male and female terminalia were examined in KOH–treated individuals. The genital structures were placed on a bed of fine glass and analyzed using an Olympus SZ61 stereomicroscope equipped with a Canon 650D camera and an LM Digital SLR Adapter (Micro Tech Lab, Austria). Layer photos were combined using the software Combine ZP (Hadley 2011). The drawings were created in Adobe Photoshop CS4 on the basis of the original sketches of Mendl (1974, *Pedicia tjederi*) and Savchenko (1978, *Pedicia semireducta*, *Pedicia persica* and *Pedicia dispar*). Freshly collected specimens were used as comparative materials, enabling the detection of distinct molecular and morphological features in the case of all three newly discovered taxa.

*Molecular techniques*. Tissue samples were collected from 152 individuals of the *Pedicia staryi* species group (83 individuals of *Pedicia staryi*, 17 of *Pedicia apusenica*, 9 of *Pedicia lobifera*, 6 of *Pedicia spinifera* and 37 of *Pedicia straminea*) and deposited in 96 well plates containing 30 μl of 96% ethanol. Four species (*Pedicia littoralis* Meigen, 1804, *Pedicia riedeli* Lackschewitz, 1940, *Pedicia nielseni* Slipka, 1955 and *Pedicia zernyi* Lackschewitz, 1940) representing the *littoralis* species group were used as outgroups for this study. The molecular processing—DNA extraction, PCR amplification, gel electrophoresis for PCR product checking, PCR cycle sequencing and sequencing—of 126 specimens and of the outgroup species was done at the Canadian Centre for DNA Barcoding (Ivanova et al. 2006, 2012, Ivanova and Grainger 2007, 2012). Specimen collection data, photographs, sequences, PCR, sequencing primers, and trace files are available through the Barcode of Life Data Systems (BOLD; Sujeevan and Hebert 2007) under the project name Tipuloidea of Europe [EUTIP]. An additional 26 individuals of *Pedicia staryi* were processed at the Interdisciplinary Research Institute on Bio–Nano–Sciences of Babeș–Bolyai University. Genomic DNA was extracted using a commercial kit (Qiagen, DNeasy Blood and Tissue Kit) and in accordance with the protocols provided by the manufacturer. The mitochondrial cytochrome c oxidase subunit I(COI) sequences were amplified using the standard LCO1490 and HCO2198 primer pair (Folmer et al. 1994). PCR was performed in a volume of 50 µl reaction mixture at 47 °C. PCR products were visualized on a 1% agarose gel and purified with a commercial kit (Promega, Wizard SV Gel and PCR Clean–Up System, USA). Sequencing was performed by Macrogen Inc. (Korea). Sequences were verified at the NCBI website using a Basic Local Alignment Search Tool (BLAST) (Johnson et al. 2008) and deposited in GenBank (accession numbers KT983903 to KT983910).

The sequences were downloaded and aligned using Clustal W in MEGA6 (Tamura et al. 2013). Phylogenetic reconstructions were performed on the haplotype dataset using a Maximum Likelihood (ML) and a Bayesian inference (BI) algorithm assuming a General Time Reversible model with a gamma-distributed variation rate across sites (G). The ML tree was estimated with Seaview, version 4 (Gouy et al. 2010) and the BI was implemented in MrBayes version 3.2.2 (Ronquist et al. 2012). Intra– and interspecific Kimura–two parameter (K2P) distance was calculated in MEGA6 (Tamura et al. 2013).

## Results

**Figure 1.**
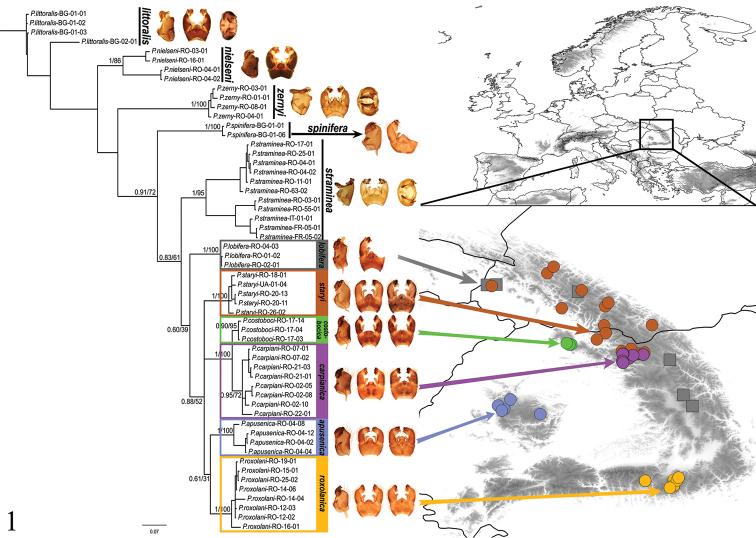
Bayesian inference (BI) tree with hypopygium profiles of species. Posterior probabilities (PP) and bootstrap values (BP, %) for the nodes are shown under the branches. Carpathian endemic species of *staryi* group are mapped. Color codes: *Pedicia lobifera* (gray square), *Pedicia staryi* (brown point), *Pedicia apusenica* (blue point), *Pedicia carpianica* (purple point), *Pedicia costobocica* (green point), *Pedicia roxolanica* (orange point).

The COI alignment of the five species belonging to the *Pedicia staryi* species group was represented by 44 haplotypes. The ML and BI analysis resulted in congruent tree topologies with high posterior probability (PP) and bootstrap (BP) values, showing a clade that includes four well-differentiated lineages of *Pedicia staryi* (Fig. 1). The K2P distance between *Pedicia lobifera*, *Pedicia apusenica*, *Pedicia staryi* and the three discovered cryptic groups ranged between 4.90% (between the cryptic groups from the Gutâi Mountains and from the southern slopes of the Rodnei Mountains) and 11.59% (between *Pedicia lobifera* and the group from the southern slopes of the Rodnei Mountains), with a mean value of 8.94%, which corresponds to the interspecific divergences generally used for molecular taxonomy of Diptera (Renaud et al. 2012, Ashfaq et al. 2014) (Table 1).

**Table 1.** Pairwise K2P distances between species within *Pedicia staryi* species group.

	*Pedicia straminea*	*Pedicia spinifera*	*Pedicia lobifera*	*Pedicia apusenica*	*Pedicia roxolanica*	*Pedicia staryi*	*Pedicia costobocica*
***Pedicia spinifera***	16,2						
***Pedicia lobifera***	12,8	12,4					
***Pedicia apusenica***	12	11,8	9,61				
***Pedicia roxolanica***	11,5	12,5	9,82	6,16			
***Pedicia staryi***	13,2	13,1	11,4	6,1	6,85		
***Pedicia costobocica***	13,3	13,4	11,2	9,25	8,58	9,05	
***Pedicia carpianica***	14	13,4	11,6	8,94	8,77	9,34	4,91

### Pedicia (Crunobia) apusenica

Taxon classificationAnimaliaDipteraPediciidae

Ujvárosi & Starý 2003 redescription

BOLD accession number: EUTIP718 to 720 and EUTIP725

Figs 2 , 3 , 4 , 5

#### Type material.

Holotype male and three paratype males collected in Romania, Apuseni Mountains, Padiș Protected Area, 1 km west of Poiana Vărășoaia, near the Cetatea Rădesei Cave, 1320 m, 46°37.806'N, 22°42.486'E, 21.July 1999, leg. L. Ujvárosi (L. Keresztes). The holotype (CN: TI96) and paratypes (CN: TI97, TI98, TI99) are deposited in the Museum of Zoology of the Babeș–Bolyai University (MZBBU), Cluj Napoca, Romania.

#### Other material.

Romania: Apuseni Mountains, Padiș Protected Area, 1 km west of Poiana Vărășoaia, near the Cetatea Rădesei Cave, 1320 m, 46°37.800'N, 22°42.480'E, 03.Aug.2003, 9 ♂♂, leg. L. Keresztes, 20.Aug.2013, 12 ♂♂, leg. A.L. Dénes; Apuseni Mountains, Stâna de Vale, 1140 m, 46°41.250'N, 22°36.546'E, 19.Aug.2013, 1♂ 1♀ leg. A.L. Dénes; Apuseni Mountains, 2 km west of Rogojel, 1290 m, 22°48.528'E, 22°48.528'E, 14.July.2014, 3 ♂♂ leg. L.P. Kolcsár; Apuseni Mountains, Boga, Boga Valley, 700 m, 46°36.576'N, 22°40.674'E, 15.Aug.2015, 1 ♂, leg. E. Török. Materials are stored in 96% ethanol or pinned dry and are deposited in the Diptera Collection of the Faculty of Biology and Geology, Cluj-Napoca, Romania

#### Diagnosis.

The species is distinguished from all other species of the *Pedicia staryi* group by the following combination of characteristics: all flagellomeres are almost uniformly colored, and there are no dark lines between antennae; the abdominal stripe starts from the second segment; the tip of the last palpus segment is darker than other segments; 9^th^ tergite has a rounded median lobe, with a small apical emersion.

#### Redescription.

**Figures 2–9.**
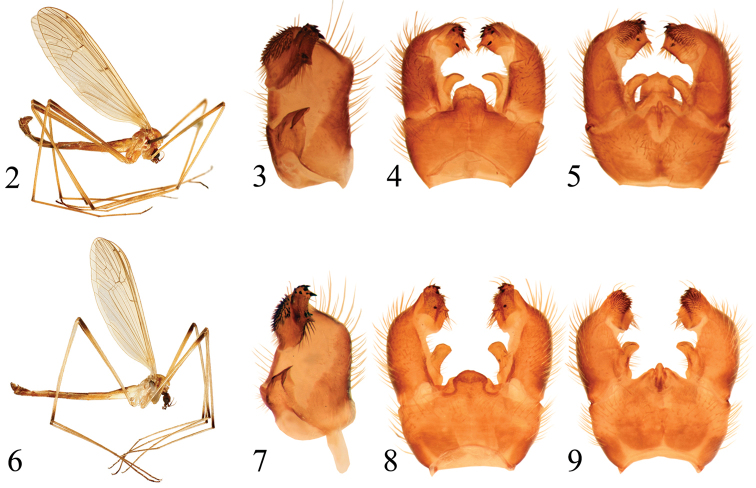
**2–5** *Pedicia apusenica* male: **2** lateral habitus **3** inner lateral view of the gonocoxite **4** male hypopygium dorsal view **5** male hypopygium ventral view **6–9** *Pedicia roxolanica* sp. n. holotype male: **6** lateral habitus **7** inner lateral view of the gonocoxite **8** male hypopygium dorsal view **9** male hypopygium ventral view.

Large species of a yellowish orange color (Fig. 2). Male body length is 14–15 mm, (mean 14 mm, n=20), wing length 13–15 mm (mean =14.1 mm, n=20), antenna 1.9–2.1 mm (mean 1.95, n=9). Head with light brown vertex. The antenna is 16–segmented; scape and pedicel are light brown, flagellomeres are almost uniformly yellowish. Scape is cylindrical, approximately 1.8 times longer than it is wide, pedicel oval. Flagellomere 1 (f1) and f2 are fused in some cases, first segment 2–2.5 times longer than it is wide (or 1.2 times), f2–f6 are approximately oval, f7–f14 are fusiform or cylindrical. All flagellomeres are equipped with 4–6 black bristles, about half as long as the flagellomeres. Palpus is 5–segmented; the first segment extremely short, second three palpomeres are dark brown, the ventral part is lighter and more membranous than dorsal parts; the last palpomere is dark brown only at the tip, the remaining parts are yellowish, membranous. Dorsal and lateral parts of thorax are yellowish orange. Scutum is yellow to orange, with two longitudinal lines of setae. Wing venation is yellowish brown. Pterostigma is light brown, more or less distinct. Small spots are present at Sc2, at base of Rs, at fork of Rs (mostly around r–m) and around R2 (Fig. 2). Halters have yellowish stem and dark orange to light brown knob (uniform yellowish orange in the case of specimens stored in alcohol). Legs have femora and tibiae are yellowish brown, black at the tip. Tarsi are light brown to dark brown. First abdominal segment is yellowish orange. A dark brown longitudinal stripe starts dorsally from the second abdominal segment, which widens through the 7^th^ and 8^th^ tergite and covers it. Sternites are yellowish orange to light brown, anterior sternites are lighter than caudal. Male terminalia is considerably broad (Figs 4, 5). The 9^th^ tergite is in some case darker than the remaining parts of the hypopygium. Posterior margin of 9^th^ tergite has a rounded median lobe, with a small apical top. Gonocoxite is stout, cylindrical and truncated at distal end. A flat spoon-like extension is present at the distal end of gonocoxite ventrally, directed inwards, densely covered with short black spinules (Fig. 3). Gonostylus is subterminal, inserted laterally at distal inner side of the gonocoxite and forming a nearly right angle with the long axis of the latter. Gonostylus is generally quadrangular in dorsal or ventral views, with 7–10 strong black spines mostly situated at the outer distal margin and with short slender projection at the lower (caudal) margin distally. Interbase is simple, broadened and rounded distally. **Female** has a body length of 15 mm; wing length 9 mm. Head is uniformly yellowish orange. Pedicel and scape are yellowish orange. Flagellomeres absent. Palpus is uniformly yellowish orange. Dorsal and lateral parts of the thorax are uniformly yellowish. Legs have coxae and trochanters yellowish orange. Wings are partly reduced, and the females are flightless. Venation is yellowish orange, having no spots on the wing. Abdomen is uniformly light orange. Female terminalia has a wide cercus, dagger-like, and raised upward at the tip. Both Hypovalvae are wide, darker than the tenth sternite. At the dorsal margin, there are seven pairs of curved, strong, needle–like setae. One seta is separated distally from the rest. A strong sensory seta is visible on the distal part of the hypovalvae, which extends beyond the end of hypovalvae.

### Pedicia (Crunobia) roxolanica

Taxon classificationAnimaliaDipteraPediciidae

Kolcsár, Keresztes & Dénes sp. n.

http://zoobank.org/69D3AFD5-E970-4429-8C00-98B08789E01A

GenBank accession number: KT983903; BOLD accession number: EUTIP669, EUTIP670, EUTIP691 to 694 and EUTIP710

Figs 6 , 7 , 8 , 9 , 25 , 26

#### Type material.

Holotype male and two paratype males collected in Romania, Baiu Mountains, Azuga, Limbășel Valley, 1200 m, 45°29.574'N, 25°35.910'E, 26.Aug.2014, L.P. Kolcsár. The pinned dry holotype (Catalog Number–CN: TI101) and paratypes (CN: TI102, TI103) are deposited in the Museum of Zoology of the Babeș–Bolyai University (MZBBU), Cluj Napoca, Romania.

#### Other material.

Romania: Bucegi Mountains, Sinaia, Cota 1400, 1400 m, 45°21.258'N, 25°31.278'E, 21.July.2004, 4 ♂♂, leg. L. Keresztes; Iezer–Păpușa Mountains, Lerești, Voina Hut, 970 m, 45°26.526'N, 25°2.670'E, 03.Aug.2006, 4 ♂♂, leg. L. Keresztes; Bucegi Mountains, Sinaia, Peleș Valley, 1300 m, 45°22.092'N, 25°30.978'E, 04.Aug.2006, 1 ♂, leg. L. Keresztes; same site, 26.July. 2013, 6 ♂♂, leg. L. Keresztes and Á. Péter; Baiu Mountains, Azuga, Casariei Valley, 1025 m, 45°26.868'N, 25°34.260'E, 20.June.2013, 1 ♂, leg. E. Török & L. Keresztes; Bucegi Mountains, Moroeni, Dichan Hut, 1575 m, 45°19.506'N, 25°27.294'E, 26.July.2013, 1 ♂, leg. L. Keresztes & Á. Péter; Baiu Mountains, Azuga, Limbășel Valley, 1200 m, 45°29.574'N, 25°35.910'E, 26.Aug.2014, 3 ♂♂, leg. L.P. Kolcsár; Bucegi Mountains, Moroeni, Cheile Orzei, 1366 m, 45°17.682'N, 25°25.434'E, 18.Aug.2015, 2 ♂♂, leg. L. Keresztes. All materials are stored in 96% ethanol and deposited in the Diptera Collection of the Faculty of Biology and Geology, Cluj-Napoca, Romania.

#### Etymology.

The species is named after an ancient population from the southern border of the Carpathians, suggesting its ancient origin, which is revealed by deep genetic and morphological structuring.

#### Diagnosis.

The new species is distinguished from all other species of the *Pedicia staryi* group by the following combination of characteristics: all flagellomeres are almost uniformly colored; the abdominal stripe starts from the first segment; the tip of last palpus segment is the same color as the other segments; 9^th^ tergite has a rounded or rarely five angled median lobe, with a notch on the tip or rarely with a small apical emergence.

#### Description.

Large species of a yellowishorange color (Fig. 6). Males body length is 13–15 mm, (mean 14.2 mm, n=7), wing length 13–14.5 mm (mean =13.9 mm, n=7), antenna 1.6–1.9 mm (mean 1.7, n=5). The head has vertex yellowish orange to dark brown (Fig. 25). Antenna is 13–15 segmented; scape and pedicel are yellowish brown to light brown, flagellomeres are almost uniformly yellowish brown. Scape is cylindrical approximately 2 times longer than width, pedicel wider apically than basally, 1.2–1.5 times longer than width in middle. F1 and f2 fused in some cases, then first segment 2 times longer than width (else 1.2 times), f2–f6 approximately oval, f7–f12 fusiform, last one or two segments elongated or orb-like. All flagellomeres with 4–6 black bristles, about half as long as the flagellomeres. Palpus 5–segmented; 2–4 palpomeres dark brown, the ventral parts lighter and more membranous than dorsal parts; the basal part of the last palpomere is light brown, dark brown at tip. Rostrum is slightly darker than vertex, margin of the labellum brown. Lateral parts of the thorax are yellowish orange, only the front margin of the katepisternum is light brown in some cases. Scutum is dark orange, with two longitudinal hair lines. Center of postnotum is yellowish orange, lateral margin is dark orange to light brown. Wing venation is yellowish brown (Fig. 6). Pterostigma is light orange, less visible. Small spots are present at Sc2 and around R2, spots are not visible around r–m and base of Rs, only the venation is slightly darkener. Halters have yellowish stem and orange knob. The legs femora is light brown and tibiae yellowish brown, and both are black at tips. Tarsi are light brown to dark brown. Dorsally on the abdomen is a pale to dark brown longitudinal stripe starting from the first abdominal segment. The 7^th^ and 8^th^ sternites and tergites are light brown. Male terminalia is considerably broad (Figs 8, 9). The 9^th^ tergite is generally darker than the remaining parts of the hypopygium. Posterior margin of 9^th^ tergite has a rounded or rarely five angled median lobe, with a notch on the tip or rarely with a small apical emergence. Gonocoxite is stout, cylindrical, and truncated at distal end (Fig. 7). Flat spoon-like extension is present at the distal end of gonocoxite ventrally, directed inwardly, covered densely in short black spinules and partly hides the gonostylus in ventral view. Gonostylus is subterminal, inserted laterally at distal inner side of the gonocoxite, forming a nearly right angle with the latter. Gonostylus is generally quadrangular in dorsal or ventral views, with 7–11 strong black spines mostly situated at outer distal margin and with short slender projection at lower (caudal) margin distally, which is less conspicuous in some cases. Interbase simple, broadened and rounded distally.

**Female** is unknown.

### Pedicia (Crunobia) costobocica

Taxon classificationAnimaliaDipteraPediciidae

Kolcsár, Keresztes & Dénes sp. n.

http://zoobank.org/98237E45-3EF8-4F65-8462-1087DA5DFEB1

BOLD accession number: EUTIP695, EUTIP698 and EUTIP708

**Figures 10–17.**
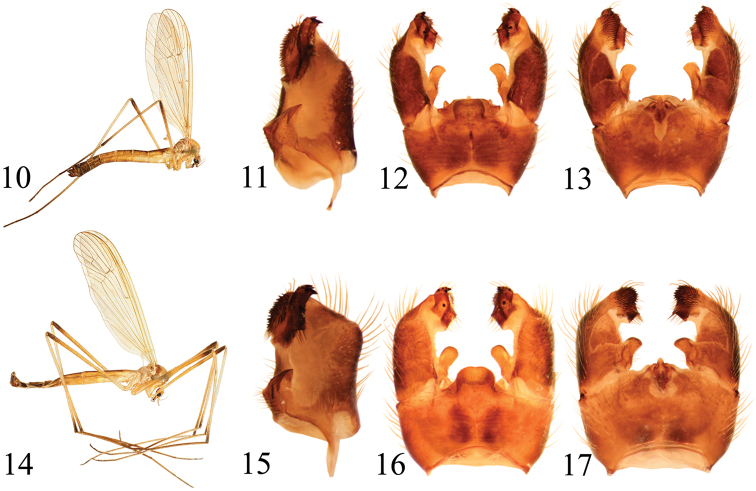
**10–13** *Pedicia costobocica* sp. n. holotype male: **10** lateral habitus **11** inner lateral view of the gonocoxite **12** male hypopygium dorsal view **13** male hypopygium ventral view **14–17** *Pedicia carpianica* sp. n. holotype male: **14** lateral habitus **15** inner lateral view of the gonocoxite **16** male hypopygium dorsal view **17** male hypopygium ventral view.

Figs 10 , 11 , 12 , 13

#### Type material.

Holotype male and paratype male collected in Romania, Gutâi Mountains, Baia Sprie, Arinieși brook, 1015 m, 47°43.068'N, 23°44.628'E, 15.May.2014, leg. L.P. Kolcsár. The pinned dry holotype (CN: TI104) and paratype (CN: TI105) are deposited in the Museum of Zoology of the Babeș–Bolyai University (MZBBU), Cluj Napoca, Romania.

#### Other material.

Romania: Gutâi Mountains, Baia Sprie, Gutâi Pass, 1070 m, 47°41.898'N, 23°49.128'E, 26.May.2012, 2 ♂♂, leg. E. Török, L.P. Kolcsár & L. Keresztes; Gutâi Mountains, Baia Sprie, Gutâi Pass, 47°41.634'N, 23°47.226’, 15.May.2013, 1 ♂ 1 ♀, E. Török & L. Keresztes; Gutâi Mountains, Baia Sprie, Arinieși brook, 1015 m, 47°43.068'N, 23°44.628'E, 15.May.2014, 7 ♂♂, leg. L.P. Kolcsár. All material is stored in 96% ethanol and deposited in the Diptera Collection of the Faculty of Biology and Geology, Cluj-Napoca, Romania.

#### Etymology.

The species is named after an ancient population from the northern part of the Eastern Carpathians suggesting its ancient origin revealed by deep genetic and morphological structuring.

#### Diagnosis.

The new species is distinguished from all other species of the *Pedicia staryi* group by the following combination of characteristics: the last 1–2 antennal segments are darker than others; black line between antennae; the abdominal stripe starts from the second segment; mean body size reach 13 mm; pedicel and scape darker than first flagellomere, face dark brown; 9^th^ tergite has a rounded median lobe, usually with a notch on the tip.

#### Description.

Medium sized species of a yellowish orange color (Fig. 10). **Male** body length is 10–14 mm, (mean 12.9 mm, n=8), wing length 11–14.5 mm (mean =13 mm, n=8), antenna 1.7 mm (mean 1.7, n=5). The head has vertex dark orange to light brown, the frontal part is brown mostly around the antennas and a narrow dark line is present between antennae. Antenna is 15–16 segmented; scape and pedicel is light brown, flagellum is almost uniformly yellowish, only the last 1–2 segments are darker. Scape is cylindrical approximately 2 times longer than width; pedicel is wider apically than basally, 1.2–1.5 times longer than width in the middle. First flagellomere is 1.3–1.4 times longer than width, flagellomeres 2 to 11 are approximately oval, apical flagellomeres are more elongated. Border between flagellomeres 12 and 13 is less distinct, frequently merge together; flagellomere 13 is elongated. All flagellomeres have 4–6 black bristles, about half as long as flagellomeres. Palpus is 5–segmented; 2–4 palpomeres are dark brown, the ventral parts are lighter and more membranous than dorsal parts; the last palpomere is dark brown only in the basal part and at tip. Rostrum is slightly darker than vertex, margin of the labellum is brown. Dorsal and lateral parts of thorax are yellowish orange. Scutum is orange, with two yellowish longitudinal lines of setae. Wing has yellowish brown venation (Fig. 10). Pterostigma is light orange, less visible. Small spots are present at Sc2, at base of Rs, around r–m and around R2. Halters with yellowish stem and dark orange knob. Legs have femora light brown and tibiae yellowish brown, and both are black at the tip. Tarsi are light brown to dark brown. First abdominal segment yellowish orange, sometimes the posterior margin brown. Dark brown longitudinal stripe in the abdominal tergum starts from the second segment. The 7^th^ and 8^th^ sternites and tergites are dark brown. The male terminalia is considerably broad (Fig. 12, 13). The 9^th^ tergite is generally darker than the remaining parts of hypopygium. Posterior margin of 9^th^ tergite has a rounded median lobe, usually with a notch on the tip. Gonocoxite is stout, cylindrical and narrowing at distal end (Fig. 11). A flat spoon-like extension is present at the distal end of gonocoxite ventrally, directed inwards, densely covered with short black spinules. Gonostylus is subterminal, inserted laterally at the distal inner side of gonocoxite, forming a nearly right angle with the latter. Gonostylus is generally quadrangular in dorsal or ventral views, with 6–9 strong black spines mostly situated at outer distal margin and distally with short slender projection at lower (caudal) margin, which is less conspicuous in some cases. Interbase is simple, broadened and rounded distally.

**Female** has a body length of 12 mm, wing length 11 mm, antenna 1.6 mm. General color is yellowish. Head is dark orange, frontal part is light brown mostly around the antennas, a narrow dark line is present between antennas. Pedicel and scape are dark orange. The antenna has 14 yellowish orange flagellomeres. Palpus is uniformly yellowish orange. The dorsal and lateral parts of the thorax are yellowish. Legs have coxae and trochanters yellowish orange. Wings are well-developed, having the ability to fly. Wing venation is yellowish orange, small spots are present at Sc2, and at around r–m. Abdomen is uniformly light orange. Female terminalia has wide cercus, dagger-like, with tip raised upward. Hypovalvae are each wide, darker than the tenth sternite with nine pairs of curved knitting needle-like strong setae at the dorsal margin. The distal seta is isolated from the rest. Two pairs of sensory setae are visible at the end of the hypovalvae, and they extend beyond the end of hypovalvae.

### Pedicia (Crunobia) carpianica

Taxon classificationAnimaliaDipteraPediciidae

Kolcsár, Keresztes & Dénes sp. n.

http://zoobank.org/3180ABB8-A7CC-4A56-9ABA-BEF6E119C821

Gen Bank accession number: KT983904 to KT983906; BOLD accession number: EUTIP095, EUTIP096, EUTIP475, EUTIP478 and EUTIP480

Figs 14 , 15 , 16 , 17 , 27

#### Type material.

Holotype male and two paratypes males collected in Romania, Rodnei Mountains, Anieș, Tomnaticul Valley, 700 m, 47°27.768'N, 24°45.696'E, 19.Aug.2014, leg. L.P. Kolcsár. The pinned dry holotype (CN: TI106) and paratypes (CN: TI107, TI108) are deposited in the Museum of Zoology of the Babeș–Bolyai University (MZBBU), Cluj Napoca, Romania.

#### Other material.

Romania: Rodnei Mountains, Rodna, Vinului Valley, 1000 m, 47°30.918'N, 24°50.094'E, 15.Aug.2008, 2 ♂♂, leg. L. Keresztes; Rodnei Mountains, Cormaia, Cormaia Valley, 750 m, 47°26.328'N, 24°39.702'E, 26.Aug.2010, 11 ♂♂, leg. R. Vaida; Rodnei Mountains, Anieș, Izvorul Mare Valley, 1220 m, 47°32.274'N, 24°40.362'E, 28.July.2011, 1 ♂, leg. R. Vaida; Rodnei Mountains, Anieș, Cepelor Spring, 1165 m, 47°31.404'N, 24°45.024'E, 19.Aug.2014, 3 ♂♂ leg. L.P. Kolcsár; Rodnei Mountains, Anieș, Tomnaticul Valley, 700 m, 47°27.768'N, 24°45.696'E, 19.Aug.2014, 11 ♂♂ leg. L.P. Kolcsár; Rodnei Mountains, Valea Mare, Rotunda Pass, 1165 m, 47°31.812'N, 25°0.810'E, 19.Aug.2014, 1 ♂, leg. L.P. Kolcsár. Material is stored in 96% ethanol or pinned dry and deposited in the Diptera Collection of the Faculty of Biology and Geology, Cluj-Napoca, Romania.

#### Etymology.

The species is named after an ancient population from the Eastern Carpathians suggesting its ancient origin revealed by deep genetic and moprhological structuring.

#### Diagnosis.

The new species is distinguished from all other species of the *Pedicia staryi* group by the following combination of characteristics: the last 1–2 antennal segments are darker than the others, black line between antennae; the abdominal stripe starts from the second segment; mean body size reaches 15.4 mm; pedicel and scape have the same color as the first flagellomeres; only the lump is darker than other parts of the head; 9^th^ tergite has a rounded or five angled median lobe, usually with a notch on the tip.

#### Description.

Large species with yellowish orange color (Fig. 14). **Male** body length is 13–17 mm, (mean 15.4 mm, n=13), wing length 13.5–17 mm (mean =15.4 mm, n=13), antenna 1.9–2.1 mm (mean 1.98, n=7). Head has vertex light brown with light yellow setae. Rostrum is slightly darker than vertex, margin of the labellum is dark brown, a narrow dark (grayish brown in the case of dry specimens) line present between antennas. Antenna is 14–16 segmented, almost uniformly yellowish, only the last 2–3 segments are darker. Scape is cylindrical, approximately 2 times longer than width, pedicel slightly wider apically than basally, 1.2–1.5 times longer than its width in the middle. First flagellomere (f1) fusiform, f2–f11(13) oval, only the last (f14) is cylindrical. All flagellomeres with 4–6 black bristles, about half as long as the flagellomeres. Palpus is 5–segmented, 2–4 palpomeres are dark brown, the ventral parts lighter and more membranous than the dorsal parts; the last palpomere dark brown at the base and tip. The dorsal and lateral parts of the thorax are yellowish orange. Scutum is orange, with two yellowish longitudinal lines of setae. In few cases the lateral margin of the postnotum is dark orange or light brown. Wing venation is yellowish brown (Fig. 14). Pterostigma is light orange, less conspicuous. Small spots present around Sc2, at the base of Rs, r–m and around R2 and R1, but in some cases less visible at the end of R1, only the venation is darker. Halters have yellowish stem and dark orange knob. Legs have light brown femora and yellowish brown tibiae, and are both black at the tip. Tarsi are light brown to dark brown. First abdominal segment yellowish orange, sometimes the posterior margin brown (Fig. 27). The dark brown dorsal longitudinal stripe on the abdomen starts from the second segment (Fig. 27). The 7^th^ and 8^th^ sternites and tergites are dark brown. Male terminalia is considerably broader (Figs 16, 17). The 9^th^ tergite generally darker than the remaining parts of hypopygium. Posterior margin of 9^th^ tergite has a rounded or five angled median lobe, usually with a notch on the tip. Gonocoxite is stout, cylindrical, and truncated at the distal end (Fig. 15). A flat spoon-like ventral extension is present at the distal end of gonocoxite, directed inwardly and densely covered with short black spinules. Gonostylus has subterminal position, inserted laterally at the distal inner side of gonocoxite and forming a nearly right angle with the long axis of the latter. Gonostylus is generally quadrangular in dorsal or ventral view, with 6–9 strong black spines mostly situated at the outer distal margin and with a short slender projection at the lower (caudal) margin distally. Interbase is simple, broadened and rounded distally.

**Figures 18–27.**
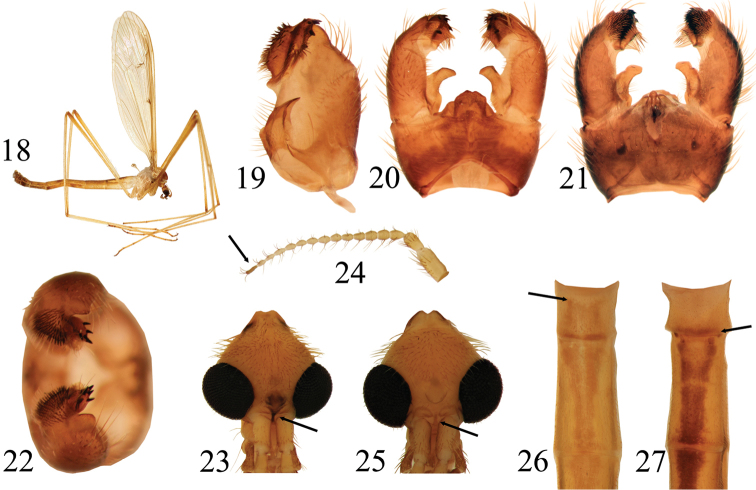
**18–24** *Pedicia staryi* male: **18** lateral habitus **19** inner lateral view of the gonocoxite **20** male hypopygium dorsal view **21** male hypopygium ventral view **22** male hypopygium caudal view **23** head dorsal view **24** antenna **25**–**26** *Pedicia roxolanica* sp. n.: **25** head dorsal view **26** dorsal view of the cranial part of abdomen **27** *Pedicia carpianica* sp. n.: dorsal view of the cranial part of abdomen.

**Female** is unknown.

### Pedicia (Crunobia) staryi

Taxon classificationAnimaliaDipteraPediciidae

Savchenko, 1978 redescription

Gen Bank accession number: KT983907 to KT983910; BOLD accession number: EUTIP709

Figs 18 , 19 , 20 , 21 , 22 , 23 , 24

#### Type material.

Holotype male Ukraine: Ivano–Frankivsk Oblast, environs settlements Vorokhta, Verkhovinsk region, 18.VI 1962 leg. E.N Savtshenko (Savchenko); deposited in the National Museum of Natural History, Kiev, Ukraine. Based of photos taken by Valery A. Korneyev.

#### Other material.

Ukraine: Gorgan Mt., Bukovel, 1120 m, 48°23.340'N, 24°26.010'E, 29.July.2014, 21 ♂♂ 1 ♀, leg. E. Török & L.P. Kolcsár. Romania: Rodnei Mountains, Borșa, Cailor Waterfall, 1700 m, 47°35.292'N, 24°39.510'E, 16.June.2010, 1 ♂, leg. R. Vaida; Maramureș Mountains, Borșa, Vișeu River, 1000 m, 47°37.374'N, 24°48.582'E, 17.May.2013, 1 ♂ leg. E. Török; Rodnei Mountains, Gura Lalei, Lalei Valley, 1200–1800 m, 47°32.070'N, 24°54.930'E, 20.July.2013, 13 ♂♂, leg. L. Keresztes & L.P. Kolcsár; Maramureș Hills, Leordina, 490 m, 47°46.752'N, 24°14.574'E, 17.May.2014, 3 ♂♂, leg. L.P. Kolcsár; Maramureș Mountains, Repedea, Repedea Valley, 790 m, 47°53.202'N, 24°23.418'E, 17.May.2014, 20 ♂♂ 1 ♀, leg. L.P. Kolcsár; Maramureș Mountains, Gura Lalei, Bistrița River, 1025 m, 47°33.858'N, 25°1.800'E, 20.Aug.2014, 5 ♂♂ leg. L.P. Kolcsár. Material stored in 96% ethanol or pinned dry and is deposited in the Diptera Collection of the Faculty of Biology and Geology, Cluj-Napoca, Romania.

#### Diagnosis.

The species is distinguished from all other species of the *Pedicia staryi* group by the following characters combination: the last 1–3 antennal segments are darker than others; black line between antennae; two brown spots on the scutum; abdominal dorsal stripe starts from first abdominal segment; 9^th^ tergite has a rounded or five angled median lobe, sometimes with a notch on the tip.

#### Redescription.

Large species having general color yellowish orange (Fig. 18). **Male** body length is 13–16 mm, (mean 14.2 mm, n=20), wing length 13–15 mm (mean =14.1 mm, n=20), antenna 1.9–2.1 mm (mean 1.95, n=9). Head has vertex light brown (specimens from Maramureș Mountains and Rodnei Mt.) or yellowish orange (specimens from Gorgan Mt.), with rostrum light brown to brown mostly around the antennas, a narrow dark (greyish brown in the case of dry specimens) line present between antennas (Fig. 23). Antenna is 16–segmented; almost uniformly yellowish, only the last 1–3 segments are darker (Fig. 24). Scape is cylindrical approximately 2.5 times longer than wide; pedicel is little wider apically than basally, 1.2–1.5 times longer than wide in the middle. First flagellomere (f1) is narrower than f2, f2–f6 are approximately ovals, f7–f12 are fusiforms, f13–14 cylindricals. All flagellomeres have 4–6 black bristles, about half as long as the flagellomeres. Palpus is 5–segmented; 2–4 palpomeres are dark brown, the ventral parts are lighter and more membranous than dorsal parts; the last palpomere is dark brown in the basal and at the tip. Lateral parts of the thorax are yellowish orange, only the frontal margin of the katepisternum is light brown in some cases. Scutum is dark orange, with two longitudinal lines of setae. Savtshenko (1978) mentions in the original description of the species the presence of an ochre yellow longitudinal stripe on the prescutum. However, this feature is visible only in some dry preserved specimens. Two diffuse dark orange to brown spots are presents near the scutellum. Postnotum is yellowish orange with an oval brown spot, which is lighter in the middle and sometimes a light line separates it in two parts. Wing has yellowish brown venation (Fig. 18). Pterostigma is light orange, less conspicuous. Small spots are present at Sc2, at base of Rs, at fork of Rs (mostly around r–m) and around R2. The spots at the base of Rs and around r–m are less visible, only the venation is darker. Halters stems are yellowish and knobs are dark orange (uniform yellowish orange in case of specimens stored in alcohol). Legs have femora dark orange to light brown and tibiae are yellowish brown, both black at the tip. Tarsi are light brown to dark brown. A light brown to dark brown longitudinal stripe is present on the abdomen, positioned dorsally to first segment. The 7^th^ and 8^th^ sternites and tergites are dark brown. Male terminalia is considerably broader than abdominal segments (Figs 20, 21). The 9^th^ tergite is generally darker than remaining parts of hypopygium. Posterior margin of 9^th^ tergite has a rounded or five angled median lobe, sometimes with a notch on the tip. Gonocoxite is stout, cylindrical, and truncated at distal end (Fig. 19). A flat spoon-like extension is present ventrally at the distal end of gonocoxite, directed inwardly, and densely covered with short black spinules. Gonostylus is subterminal, inserted laterally at distal inner side of gonocoxite and forming a nearly right angle with the long axis of the latter. Gonostylus is generally quadrangular in dorsal or ventral views, with 5–8 strong black spines, mostly situated at the outer distal margin and with short slender projection at lower (caudal) margin distally. Interbase simple, broadened and rounded distally. **Female** body length is 16.5–17 mm, wing length 12–13 mm, antenna 1.7 mm. Head is uniformly dark orange. Pedicel, scape and flagellomeres are yellowish orange, only the last flagellomere is darker. Palpus is light brown. The dorsal and lateral parts of the thorax are yellowish. Legs have coxae and trochanters yellowish orange. Femora, tibia and tarsomeres (I–IV) are orange. Last tarsomere (V) is black. Wings are developed, and females are able to fly. Wing venation is yellowish orange, having no spots on the wing. Abdomen is dark orange, only the first and second tergite are light brown. Female terminalia has wide cerci, widest in middle and raised upward towards the tip. Hypovalvae are each wide, darker than the tenth sternite. Dorsal margin has 7–8 pairs curved knitting needle-like strong setae. One seta is distally isolated from the rest. Sensory setae are visible in hypovalvae, and they extend beyond the end of hypovalvae.

### Key to species of *Crunobia* subgenus

**Table:** 

1	Gonostylus has dorsally only two or three big thorn-like dark spines (Figs 29, 33, 40, 41)	**2**
–	Gonostylus has dorsally 5–11 claw-like dark spines (Figs 2, 7, 22, 46, 47)	(*staryi* species group) **11**
2	Gonostylus extends ventrally and forms a big obtuse projection, densely covered by black spinule (Figs 29, 31, 41, 43)	**3**
–	Gonostylus without such projection (Figs 33, 35)	**8**
3	Wings strongly reduced, much shorter than the abdomen	**Pedicia (Crunobia) semireducta Savchenko, 1978**
–	Wings well-developed and as long or sometimes longer than the abdomen (Fig. 28)	**4**
4	Gonostylus ventral projection short, not wider than gonocoxite, 9^th^ abdominal tergite with a small triangular lobe with a big notch in the middle of the distal margin (Fig. 52)	**Pedicia (Crunobia) tjederi Mendl, 1974**
–	Gonostylus ventral projection long, wider than the gonocoxite, 9th abdominal tergite is differently shaped (Figs 29, 30, 31, 41, 42, 43)	**5**
5	Gonostylus ventral projection (in caudal view) is densely covered by black spinule only in the ventral parts (Fig. 43); 9^th^ abdominal lobe without notch (Figs 41, 42)	**Pedicia (Crunobia) zangheriana Nielsen, 1950**
–	Gonostylus ventral projection (in caudal view) uniformly covered by black spinule (Fig. 31); 9^th^ abdominal lobe has a notch (Fig. 30)	**6**
6	Lateral side of scutum, prescutum and wings’ base is lighter (Fig. 28)	**Pedicia (Crunobia) pallens Savchenko, 1978**
–	Lateral side of scutum, prescutum and wings’ base is darker	**7**
7	Wings are transparent, with more or less conspicuous pattern, 9^th^ abdominal tergite is narrow, narrower at its base (Fig. 54)	**Pedicia (Crunobia) persica Alexander, 1975**
–	Wings are yellowish, only with an obscure dark pattern; 9^th^ tergite wider at its base	**Pedicia (Crunobia) zernyi (Lackschewitz, 1940)**
8	Ochre–yellow species. Femora uniformly colored, the tip of femora without dark rings (Fig. 32)	**Pedicia (Crunobia) littoralis (Meigen, 1804)**
–	Dark colored species. Femora brown, dark–brown, basally lighter (Fig. 36)	**9**
9	Thorax mainly brownish yellow, with brown or yellowish brown prescutal stripes, 9^th^ abdominal tergite lobe with a big notch (Fig. 55)	**Pedicia (Crunobia) dispar Savchenko, 1978**
–	Thorax mainly gray, with grayish–brown prescutal stripes; 9^th^ abdominal tergite lobe without a notch (Figs 38, 40)	**10**
10	Medial lobe of the 9^th^ abdominal tergite triangular, narrowed towards the tip (Figs 39, 40). Wings usually grayish	**Pedicia (Crunobia) riedeli (Lackschewitz, 1940)**
–	Medial lobe of the 9^th^ abdominal tergite hexagonal, slightly narrow at the base. Wings usually yellowish (Figs 36, 37, 38)	**Pedicia (Crunobia) nielseni (Slipka, 1955)**
11	Gonostylus extended ventrally and forms a big obtuse projection (Figs 44, 45, 46, 47) .	**Pedicia (Crunobia) straminea (Meigen, 1838)**
–	Gonostylus without this projection (Figs 3, 7, 11, 15, 19, 22, 48, 50)	**12**
12	Interbases with a spine-like outgrowth (Figs 48, 49)	**Pedicia (Crunobia) spinifera Starý, 1974**
–	Interbases without spine-like outgrowth (Fig. 4, 20, 51)	13
13	Gonocoxite has on the top a conspicuous, isolated rounded lobe (figs 50, 51)	**Pedicia (Crunobia) lobifera Savchenko, 1986**
–	Gonocoxite on the top without such rounded lobe (Figs 3, 7, 11, 15 19)	**14**
14	All flagella almost uniformly colored, none darker, no black or dark brown line between antennae (Fig. 25)	**15**
–	The last 1–3 antennal segments are darker than others (Fig. 23), black or dark brown line between antennae (Fig. 24)	**16**
15	Abdominal dorsal stripe starts from the second abdominal segment (Fig. 27); tip of the 5^th^ palpus segment darker than other segments; 9^th^ tergite has a rounded median lobe, with a small apical emersion (Figs 4, 5)	**Pedicia (Crunobia) apusenica Ujvárosi & Starý, 2003**
–	The abdominal stripe starts from the first segment (Fig. 26); tip of 5^th^ palpus segment is the same color as the other segments; medial lobe of 9^th^ abdominal tergite rounded or with a notch on the tip, or rarely with a small apical emergence (Figs 8, 9)	**Pedicia (Crunobia) roxolanica Kolcsár, Keresztes & Dénes, sp. n.**
16	Two brown spots on the scutum, abdominal dorsal stripe starts from first abdominal segment (Fig. 26)	**Pedicia (Crunobia) staryi Savchenko, 1978**
–	No brown spots on scutum, stripe starts from second abdominal segment (Fig. 27)	**17**
17	Smaller species, mean body size reach 13 mm; pedicel and scape darker than first flagellomere, face dark brown	**Pedicia (Crunobia) costobocica Kolcsár, Keresztes & Dénes, sp. n.**
–	Larger species, mean body size is 15.4 mm; pedicel and scape is the same color as the first flagellomere, only the lump is darker than other parts of the head	**Pedicia (Crunobia) carpianica Kolcsár, Keresztes & Dénes, sp. n.**

**Figures 28–35.**
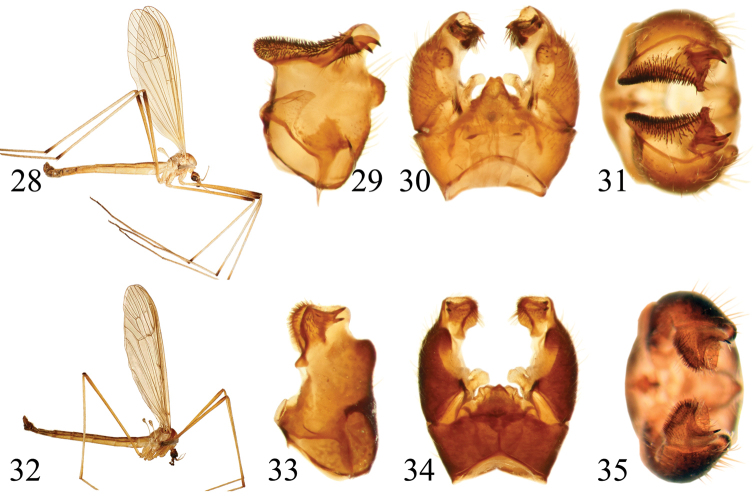
**28–31** *Pedicia pallens* male: **28** lateral habitus **29** inner lateral view of the gonocoxite **30** male hypopygium dorsal view **31** male hypopygium caudal view **32–35** *Pedicia littoralis* male: **32** lateral habitus **33** inner lateral view of the gonocoxite **34** male hypopygium dorsal view **35** male hypopygium caudal view.

**Figures 36–43.**
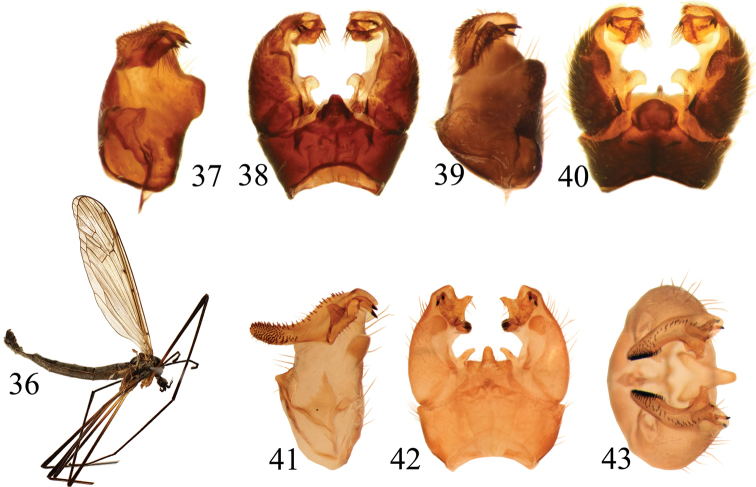
**36–38** *Pedicia nielseni* male: **36** lateral habitus **37** inner lateral view of the gonocoxite **38** male hypopygium dorsal view **39**–**40** *Pedicia riedeli*: **39** inner lateral view of the gonocoxite **40** male hypopygium dorsal view **41–43** *Pedicia zangheriana* male: **41** inner lateral view of the gonocoxite **42** male hypopygium dorsal view **43** male hypopygium caudal view.

**Figures 44–51.**
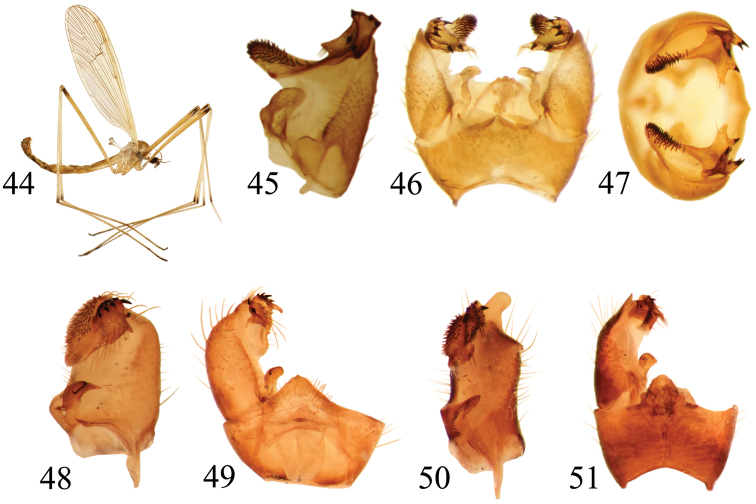
**44–47** *Pedicia straminea*: **44** male lateral habitus **45** inner lateral view of the gonocoxite **46** male hypopygium dorsal view **47** male hypopygium caudal view **48–49** *Pedicia spinifera*: **48** inner lateral view of the gonocoxite **49** male hypopygium dorsal view **50–51** *Pedicia lobifera*: **50** inner lateral view of the gonocoxite **51** male hypopygium dorsal view.

**Figures 52–55.**
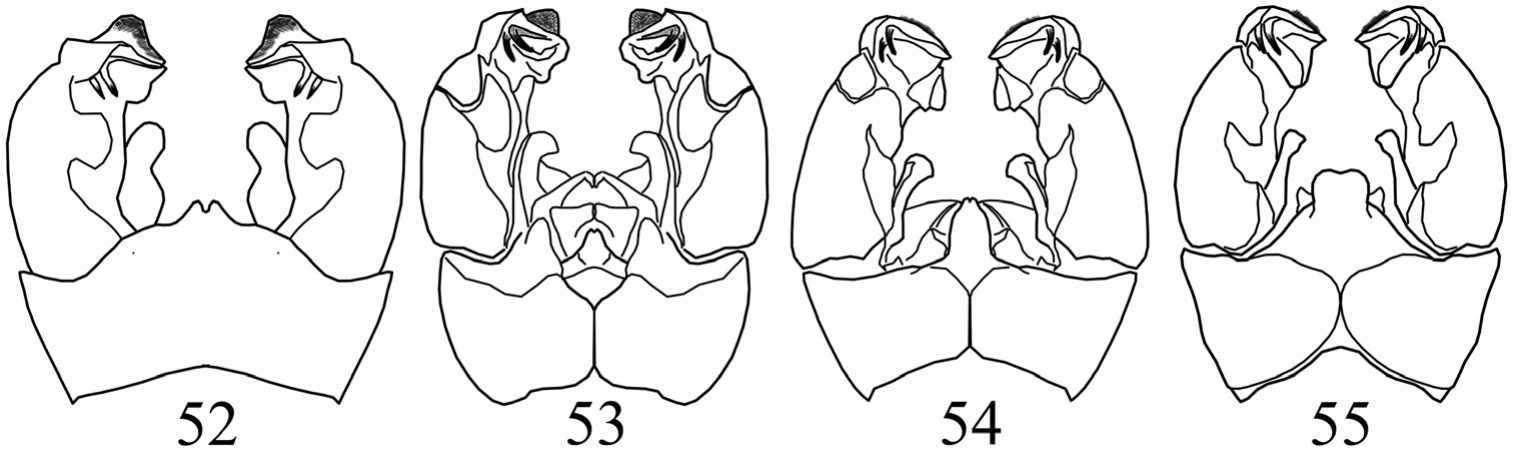
*Pedicia tjederi*: **52** male hypopygium dorsal view *Pedicia semireducta*: **53** male hypopygium dorsal view *Pedicia persica*: **54** male hypopygium dorsal view *Pedicia dispar*: **55** male hypopygium dorsal view.

## Discussion

The three newly described species, *Pedicia costobocica*, *Pedicia carpianica* and *Pedicia roxolanica* can clearly be attributed to the “*staryi*” species group *sensu* Savchenko (1986) because they have more than two black spines on the top of the gonostylus. In concordance with the previous taxonomic hypotheses, the Maximum Likelihood and Bayesian Inference phylogenetic analyses also support the *Pedicia staryi* group as a monophyletic unit. However, the six Carpathian endemics, *Pedicia apusenica*, *Pedicia lobifera*, *Pedicia staryi*, and the newly described *Pedicia costobocica* sp. n., *Pedicia carpianica* sp. n. and *Pedicia roxolanica* sp. n. form a well-defined and highly distant clade from the other two members of the group, with an average genetic distance of 12.81% in the case of *Pedicia straminea* and 12.67% in the case of *Pedicia spinifera*. Such deep genetic structures suggest autochthonous evolutionary histories with ancient divergences in the Carpathian Area (see also Dénes et al. 2015). Our genetic results are highly congruent with the morphological analyses. The newly described species are closely related to *Pedicia staryi* and *Pedicia apusenica*, by a combination of important morphological features. Morphology and genetic comparison show similar patterns. *Pedicia apusenica* and *Pedicia roxolanica* are unified in a separate group, differing from the remaining species by the uniformly colored antennae and the lack of a dark line between them. Genetic results also suggest more similarity between *Pedicia apusenica* and *Pedicia roxolanica*. *Pedicia staryi*, *Pedicia costobocica* and *Pedicia carpianica* share highly similar features, but *Pedicia staryi* is distinguished from *Pedicia costobocica* and *Pedicia carpianica* by the presence of two brown spots on the scutum and by the abdominal dorsal stripe that starts from the first segment.

The rhitral biome in Central Europe is home to an exceptionally high aquatic diversity with an important number of endemics, in contrast with the arboreal elements, which are related mostly with some classical Mediterranean core areas (Mey and Botoșăneanu 1985, Pauls et al. 2009, Bálint et al. 2011). The rithral biome of the Alps and the Pyrenees has been intensively explored over the course of the past few years; however, the Carpathians are constantly neglected, despite their basic role as important hot spots of aquatic diversity in Europe (Bálint et al. 2008, Stewart et al. 2010, Schmitt and Varga 2012, Graf et al. 2014, Török et al. 2015). The geographic projection of the newly discovered species suggests insular-like distribution in the Carpathians. The pattern is similar to the distribution of the already recognized *Pedicia apusenica*, *Pedicia staryi* or *Pedicia lobifera*, and it represents new evidence concerning the importance of the Carpathians as a significant genetic center of aquatic diversity. The northern part of the Eastern Carpathians is exceptionally rich in endemics, as suggested by a high number of other aquatic insects, such as caddisflies (Bálint et al. 2011). In our case, 4 species (*Pedicia staryi*, *Pedicia apusenica*, *Pedicia costobocica*, *Pedicia carpianica* and *Pedicia lobifera*) belonging to the *Pedicia staryi* group are exclusive inhabitants of this important northern refuge-like area, confined between the western limit of the northern Carpathians in Ukraine and the Haghimaș Mountains in Romania, where they are isolated in one or a few limited enclaves (the refugia within refugia pattern, Varga 2010). The Apuseni Mountains harbor an exceptionally high terrestrial diversity (ex. earth worms) (Csuzdi and Pop 2007), due to their isolated position from the rest of the Carpathians. The aquatic diversity however, is lower (ex. caddis flies) (Ujvárosi et al. 2008)hence they are home to only one endemic species from the group under examination: *Pedicia apusenica*. In the Southern Carpathians, the eastern part shelters a single endemic species of the group: *Pedicia roxolanica*. Despite its role as an important genetic center for a series of terrestrial (Varga 2010) and aquatic species (Bálint et al. 2009, Ujvárosi et al. 2008), the western part of the Southern Carpathians has no endemic members belonging to the *staryi* species group. Most of the newly discovered microendemic species of the Carpathians are highly specialized rithral elements concentrated near the sources of cold stenotherm springs, with an important degree of hitherto undiscovered diversity.

## Supplementary Material

XML Treatment for Pedicia (Crunobia) apusenica

XML Treatment for Pedicia (Crunobia) roxolanica

XML Treatment for Pedicia (Crunobia) costobocica

XML Treatment for Pedicia (Crunobia) carpianica

XML Treatment for Pedicia (Crunobia) staryi

## Supplementary materials

Supplementary material 1 Sampling information Data type: Collection data Explanation note: Information for sampling of the five microendemic Carpathian species (*Pedicia lobifera*, *Pedicia staryi*, *Pedicia apusenica*, *Pedicia carpianica*, *Pedicia costobocica*, *Pedicia roxolanica*), containing the name of the species, the date of collection, with localities, mountains, locations and geographic coordinates This dataset is made available under the Open Database License (http://opendatacommons.org/licenses/odbl/1.0/). The Open Database License (ODbL) is a license agreement intended to allow users to freely share, modify, and use this Dataset while maintaining this same freedom for others, provided that the original source and author(s) are credited.Avar-Lehel Dénes, Levente-Péter Kolcsár, Edina Török, Lujza Keresztes
